# Evaluation of Transparent Pressure Masks in Reducing Hypertrophic Scars: A Comparison Across Facial Zones

**DOI:** 10.1111/jocd.70860

**Published:** 2026-04-12

**Authors:** Ming Ao, Nan Chen, Bo Lu, Jianglin Tan

**Affiliations:** ^1^ Institute of Burn Research, State Key Laboratory of Trauma and Chemical Poisoning, Chongqing Key Laboratory for Disease Proteomics, Southwest Hospital Army Medical University (Third Military Medical University) Chongqing China

**Keywords:** burns, hypertrophic scar, transparent pressure mask

## Abstract

**Background:**

Facial burn scars are challenging to treat due to complex contours, and data on site‐specific efficacy of transparent pressure masks are limited.

**Aims:**

To evaluate the effectiveness of transparent pressure masks on six facial zones in treating post‐burn hypertrophic scars.

**Patients/Methods:**

We retrospectively analyzed 22 patients with post‐burn facial hypertrophic scars across six zones (mid‐forehead, bilateral zygomatic arches, bilateral cheeks, chin). Patients were treated with transparent masks and followed for 6 months. Scar assessment included Vancouver Scar Scale (VSS), thickness, hardness, coloration, pruritus, pain, and satisfaction.

**Results:**

We found that the VSS scores of the middle of forehead and bilateral zygomatic arches were significantly improved compared with scores before mask application. In the objective assessment, a significant difference in thickness of the scar was observed in the middle of forehead and chin compared with the scores before mask application. Similarly, the hardness of the scar in the chin was significantly different from that before mask application. The value of color *L* of the scar in the middle of forehead, left zygomatic arch, right cheek and chin was significantly different between baseline scores and after 6 months of wearing a transparent mask. The patients were very satisfied with the therapeutic effect. The percentage of wearing transparent mask adverse effects was 36.36%, including redness, oozing, blistering, and rash.

**Conclusions:**

Transparent pressure masks effectively improve facial burn hypertrophic scars, particularly in the mid‐forehead of forehead and bilateral zygomatic arches, and can be applied within 1 month.

## 1 Introduction

Hypertrophic scar is one of the most common cutaneous complications following burn injury, with a prevalence exceeding 70% in Asian populations [1]. Facial burns account for 47.7% of all forms of burns, second only to burns on the upper extremity areas [2]. Currently, several therapeutic options are available for the treatment of hypertrophic scar, ranging from laser therapy and steroid injections to various surgical corrections, which are aimed at improving the appearance and function of the facial hypertrophic scar. However, severe facial deformities caused by hypertrophic scars significantly complicate the process of restoring both facial appearance and function. This highlights the urgent need for developing preventive therapies. Effective preventive measures would not only inhibit scar hyperplasia and contracture, but also promote scar maturation, ultimately reducing the number and complexity of reconstructive surgeries required later. Pressure therapy has long been used as an important component of the routine treatment of hypertrophic scar. Due to the geometry of the human face, characterized by concave and convex surfaces, conventional elastic pressure garments do not provide effective pressure on the concave surface; they fail to provide an appropriate strain in the inner canthus, the nose, the nasolabial fold, and around the mouth. To address this challenge, a rigid transparent mask molded from thermoplastic materials has been proposed. Since the first fabrication of face masks using alginate impression materials, plaster bandages, and high‐temperature thermoplastic materials by Rivers et al. transparent masks have been applied as a form of pressure therapy for the prevention and treatment of facial hypertrophic scars [3]. The transparent mask fixes the face firmly and inhibits the formation of surface hyperplasia of the facial hypertrophic scar, while also minimizing the scar contracture. Moreover, considering that the material is transparent, the treatment process can be easily monitored; plastic and reconstructive surgeries can be performed at any time when necessary. However, their results were limited in whole facial hypertrophic scar. It is still unclear whether it has the same effect in the different facial zones. Therefore, this study was designed to assess the efficacy, difference, and safety of transparent pressure masks on burn patients with six facial zones including the middle of the forehead, bilateral zygomatic arches, bilateral cheeks, and chin after 6 months treatment.

## 2 Subjects and Methods

### 2.1 Data Collection

This study selected patients who underwent transparent mask from November 2020 to August 2023 from the Institute of Burn Research in the Hospital. This retrospective analysis was approved by the Ethics Committee of the Hospital. The general information of the patients (age, gender), cause of injury, depth of burn, facial healing time, and first time of wearing mask were collected. The selection criteria: (1) diagnosed with deep facial burns (burn depth of second degree (II°) and above); (2) facial hypertrophic scar within 1 year; (3) patients and their families provided informed consent and agreed to undergo the rehabilitation treatment.

### 2.2 Methods

#### 2.2.1 Screening of Patients and First Assessment

Facial scar photographs and scar characteristics were taken and assessed for all eligible patients. The features assessed included the middle of forehead, bilateral zygomatic arches, bilateral cheeks, and chin of the face, scar pruritus, pain, thickness, hardness, and coloration on 6 facial zones, as well as patient satisfaction.

#### 2.2.2 Preparation of the Transparent Mask

For eligible patients who had experienced healing of facial wounds, a certified therapist would create a transparent mask for each patient. The process involved making a negative mold first. This was achieved by taking an impression of the patient's face and ears using a special facial impression material, and then reinforcing the mold with external plaster bandages. The positive mold, used to form the final transparent pressure mask, was then created by filling the negative mold with plaster powder. Once the plaster hardened, the positive mold was removed, modified, and then molded with a high‐temperature transparent plate, and tried on before a final single‐layer transparent pressure mask was prepared for patient use. To achieve the best outcomes of the mask, patients were instructed to wear the transparent mask for a long period (> 10 h) every day, except when washing the face, applying special treatments, or occurrence of traumatic enlargement and other adverse effects. In cases requiring mask removal to release pressure, it was done within 30–60 min.

#### 2.2.3 Reassessment

In the early stage after designing the facemask (1–3 months), a therapist provided instructions to patients on proper mask wearing, facial skin cleaning, and comprehensive facial scar reduction techniques. The facemasks were adjusted in a timely manner according to changes in pressure distribution to ensure optimal wearing effects every month. The patients received reassessment about scar characteristics (pruritus, pain, thickness, hardness, coloration) and patient satisfaction in six facial zones: the middle of forehead, bilateral zygomatic arches, bilateral cheeks, and chin of the patient's face after 6 months wearing facemask by the same rehabilitation therapist.

### 2.3 Assessment Indicators

#### 2.3.1 Facial Scar Photography

Facial scar images were collected using a digital camera (Canon, EOS 6D, Canon, Japan) by taking photos of the patient's face in the front position, lateral 45° position, and lateral 90° position under the same background, distance, and light. Detailed comparisons were conducted at the end of the treatment cycle.

#### 2.3.2 Vancouver Scar Scale (VSS)

The VSS was employed to generate scores of the subjective observation of four aspects of scar pigmentation, vascularity, thickness, and pliability, with a minimum score of 0 and a maximum score of 15. The higher the score, the higher the severity of the scar.

#### 2.3.3 Scar Pruritus and Pain Score (VAS)

The VAS pain score scale is used to classify pain into 11 levels ranging from 0 to 10: with 0 denoting no pain and 10 indicating maximum pain. Based on the patient's feelings, one level is assigned between 0 and 10 to represent the degree of the pain. Scar pruritus score was determined as described above.

#### 2.3.4 Scar Thickness

The thickness of the patients' facial scar was measured using Portable Color Doppler Ultrasound System (Z6, CAN‐01002801, Shenzhen Mindray Bio‐Medical Electronics Co. Ltd., China) with higher scores indicating greater thickness.

#### 2.3.5 Shore Hardness Tester, Colorimeter

These two professional instruments were employed to objectively assess the hardness and coloration of the scar. Higher scores obtained by the durometer (Shore, HT‐6510OO, Guangzhou Landtek Instrument Co. LTD, China) indicated higher hardness. The MiniScan XE Plus spectrocolorimeter (HunterLab, Reston, VA, USA) was employed to evaluate the scar colors in terms of lightness and redness.

#### 2.3.6 Patient Satisfaction

The patients were instructed to rate their satisfaction using the comprehensive facial anti‐scarring treatment questionnaire. The higher the score, the higher the level of satisfaction.

#### 2.3.7 Assessment of Adverse Effects

The occurrence of adverse effects during the wearing period of the transparent mask, such as redness, oozing, bleeding, blistering, swelling, and rash were recorded. The assessment was conducted once a month, and each patient was followed up for at least 6 months.

### 2.4 Statistics Processing

Data analysis was performed using SPSS 23.0 statistical software, and measurement data that followed a normal distribution were expressed as M ± SD. Comparison of multiple time points in multiple groups was carried out using the repeated‐measures ANOVA, and pairwise comparisons were performed using Bonferroni's test and corrected *p*‐value, and the *p* < 0.05 was considered statistically significant.

## 3 Results

### 3.1 Demographic and Medical Characteristics of the Patients

During the study period from November 2020 to August 2023, 45 patients were collected, however due to the influence of COVID‐19 during the period of study, there are 18 patients who didn't come back for regular reassessment, which mainly leads to lose patient data. Two patient did not insist on wearing mask due to repeated scar blister, and three patients also did not wear it due to face surgery in the later stage. At last, a total of 22 patients were collected in this study. There were 14 males and 8 females in included patients. The average age was 35.95 years old (35.95 ± 16.59). The fire/flame was the first injury factor. 18 patients were the deep partial thickness burn. The average healing time was 53.00 (53.00 ± 19.48) days after burn. And the first time of wearing mask was 77.59 (77.59 ± 54.34) days after burn (Table 1).

**TABLE 1:** Demographic and medical characteristics of the patients.

Age (years)	35.95 ± 16.59
Sex	
Male	14
Female	8
Cause of injury	
Scald	2
Fire/flame	17
Electricity	1
Chemical agent	2
Other	0
Depth of burn	
Deep partial thickness burn	18
Full thickness burn	4
Facial healing time, post‐burn (days)	53.00 ± 19.48
< 30	2
30–60	11
60–90	9
First time of wearing mask, post‐burn (days)	77.59 ± 54.34
< 30	2
30–60	8
60–90	6
> 90	6

### 3.2 VSS Scores Analysis

VSS scores in the middle of the forehead and bilateral zygomatic arches were significantly improved after 6 months of wearing a transparent mask (*p* < 0.05). Although the scores of VSS in bilateral cheeks and chin exhibited a decreasing trend after 6 months, there was no significant difference (*p* > 0.05; Table 2).

**TABLE 2:** Comparison of VSS scores before and after application of masks for different sites.

Hypertrophic scar	Before treatment (M ± SD)	After 6 months (M ± SD)	*p*
Middle of forehead (VSS)	6.45 ± 3.10	4.14 ± 3.04	0.0165
Pigmentation	1.68 ± 0.84	1.00 ± 0.93	
Vascularity	1.41 ± 0.73	0.77 ± 0.81	
Pliability	1.59 ± 0.91	1.14 ± 0.77	
Height	1.77 ± 1.07	1.23 ± 0.87	
Left zygomatic arch (VSS)	8.05 ± 2.17	5.68 ± 3.09	0.0132
Pigmentation	2.14 ± 0.64	1.27 ± 0.83	
Vascularity	1.86 ± 0.47	1.23 ± 0.87	
Pliability	1.86 ± 0.64	1.55 ± 0.80	
Height	2.18 ± 0.96	1.64 ± 0.85	
Right zygomatic arc (VSS)	8.00 ± 2.09	5.77 ± 3.07	0.022
Pigmentation	2.14 ± 0.64	1.27 ± 0.83	
Vascularity	1.86 ± 0.47	1.23 ± 0.87	
Pliability	1.86 ± 0.71	1.68 ± 0.78	
Height	2.14 ± 0.89	1.59 ± 0.91	
Left cheek (VSS)	7.68 ± 2.95	6.64 ± 2.80	0.628
Pigmentation	1.95 ± 0.79	1.55 ± 0.74	
Vascularity	1.68 ± 0.65	1.32 ± 0.78	
Pliability	1.91 ± 0.87	1.86 ± 0.77	
Height	2.13 ± 1.08	1.91 ± 0.87	
Right cheek (VSS)	8.41 ± 1.82	6.72 ± 2.51	0.1251
Pigmentation	2.14 ± 0.64	1.59 ± 0.67	
Vascularity	1.86 ± 0.35	1.36 ± 0.79	
Pliability	2.09 ± 0.68	1.91 ± 0.68	
Height	2.32 ± 0.95	1.86 ± 0.83	
Chin (VSS)	8.95 ± 2.92	7.68 ± 3.41	0.3764
Pigmentation	2.23 ± 0.75	1.68 ± 0.84	
Vascularity	1.91 ± 0.61	1.50 ± 0.80	
Pliability	2.27 ± 0.98	2.18 ± 1.05	
Height	2.55 ± 1.14	2.32 ± 1.04	

*Note:* The *p* value was obtained by comparing each group after 6 months of treatment with the first treatment.

### 3.3 Scar Thickness Analysis

There were significant differences in scar thickness of the middle of forehead and chin between baseline and after 6 months of wearing a transparent mask (*p* < 0.05). The scar thickness in the bilateral zygomatic arches and cheeks was not significantly different between baseline and after 6 months of transparent mask use (*p* > 0.05; Table 3).

**TABLE 3:** Comparison of scar thickness before and after treatment for different sites.

Scar thickness (mm) (M ± SD)	Middle of the forehead	Left zygomatic arch	Right zygomatic arch	Left cheek	Right cheek	Chin
Before treatment	2.01 ± 1.21	2.25 ± 1.22	2.33 ± 1.04	2.35 ± 1.52	2.65 ± 1.28	3.74 ± 2.22
After 6 months	1.08 ± 1.01	1.46 ± 0.87	1.50 ± 0.97	1.89 ± 1.21	1.94 ± 1.09	2.47 ± 1.40
*p*	0.0375	0.1009	0.0770	0.6455	0.1682	0.0020

*Note:* The *p* value was obtained by comparing each group after 6 months of treatment with the first treatment.

### 3.4 Scar Hardness Analysis

The scar hardness in the chin was significantly different between baseline and after 6 months of wearing a transparent mask (*p* < 0.05). The scar hardness in the middle of forehead, bilateral zygomatic arches, and bilateral cheeks was not significantly different between baseline and after 6 months of wearing a transparent mask (*p* > 0.05; Table 4).

**TABLE 4:** Comparison of scar hardness before and after mask wearing for different sites.

Scar hardness (M ± SD)	Middle of forehead	Left zygomatic arch	Right zygomatic arch	Left cheek	Right cheek	Chin
Before treatment	9.85 ± 10.18	9.76 ± 9.60	9.37 ± 10.09	8.76 ± 8.93	8.10 ± 8.26	18.49 ± 3.13
After 6 months	4.60 ± 5.27	5.50 ± 5.28	5.20 ± 4.59	6.50 ± 6.45	6.79 ± 7.73	12.47 ± 9.64
*p*	0.0711	0.1983	0.2160	0.9864	> 0.9999	0.0287

*Note:* The *p* value was obtained by comparing each group after 6 months of treatment with the first treatment.

### 3.5 Scar Color Analysis

The scar color *L* value in the middle of forehead, the left zygomatic arch and the right cheek was significantly different between baseline scores and after 6 months of wearing a transparent mask (*p* < 0.05). It was also observed that scar color *L*, *b* values in the chin varied significantly between baseline and after 6 months of wearing a transparent mask (*p* < 0.05). The color of scars color *a*, *b* values on the middle of forehead, left zygomatic arch, and right cheek were not different between baseline and after 6 months of transparent mask use (*p* > 0.05). Similarly, no significant changes were observed in scar color (*L*, *a*, and *b* values) on the right zygomatic arch and left cheek during the same period (*p* > 0.05; Table 5).

**TABLE 5:** Comparison of scar color detected by spectrocolorimeter before and after mask wearing for different sites.

Scar color (M + SD)	Middle of forehead			Left zygomatic arch			Right zygomatic arch			Left cheek			Right cheek			Chin		
	*L*	*a*	*b*	*L*	*a*	*b*	*L*	*a*	*b*	*L*	*a*	*b*	*L*	*a*	*b*	*L*	*a*	*b*
Before treatment	49.18 ± 3.39	11.35 ± 2.37	11.81 ± 2.58	48.34 ± 4.04	11.84 ± 2.68	11.35 ± 2.39	47.23 ± 3.55	11.10 ± 2.15	11.68 ± 2.76	48.40 ± 3.69	10.70 ± 2.15	11.60 ± 2.63	47.34 ± 3.28	11.39 ± 2.30	11.30 ± 2.75	44.73 ± 3.15	11.98 ± 3.10	9.81 ± 3.04
After 6 months	53.68 ± 3.49	10.10 ± 2.06	13.26 ± 2.44	51.62 ± 4.29	10.16 ± 2.50	12.63 ± 3.31	51.04 ± 5.13	11.24 ± 2.33	12.08 ± 3.10	51.08 ± 3.33	10.68 ± 1.84	11.65 ± 2.59	50.63 ± 3.43	10.67 ± 2.14	12.44 ± 3.18	49.81 ± 3.71	11.91 ± 2.72	11.84 ± 3.04
*p*	0.0002	0.4382	0.2437	0.0105	0.0540	0.3708	0.2587	> 0.999	> 0.999	0.0505	> 0.999	> 0.999	0.0102	0.9277	0.5103	< 0.0001	> 0.999	0.0445

*Note:* The *p* value was obtained by comparing each group after 6 months of treatment with the first treatment.

### 3.6 VAS Scores Analysis

The VAS scar pruritus and pain scores of the middle of forehead, bilateral zygomatic arches, bilateral cheeks, and chin did not differ significantly after 6 months of wearing a transparent mask (*p* > 0.05; Table 6).

**TABLE 6:** Comparison of the VAS scar pruritus and pain scores before and after mask wearing for different sites.

VAS scores (M ± SD)	Middle of forehead		Left zygomatic arch		Right zygomatic arch		Left cheek		Right cheek		Chin	
	Pruritus	Pain	Pruritus	Pain	Pruritus	Pain	Pruritus	Pain	Pruritus	Pain	Pruritus	Pain
Before treatment	1.91 ± 2.33	0.82 ± 1.89	2.00 ± 2.60	0.73 ± 1.67	2.64 ± 2.61	0.86 ± 1.72	2.09 ± 2.56	0.73 ± 1.67	2.54 ± 2.67	0.86 ± 1.73	2.14 ± 2.56	1.36 ± 2.17
After 6 months	1.09 ± 2.02	0.00 ± 0.00	1.41 ± 2.42	1.36 ± 0.64	1.55 ± 2.42	1.36 ± 0.64	1.55 ± 2.42	0.27 ± 0.88	1.55 ± 2.42	0.27 ± 0.88	2.00 ± 2.56	0.45 ± 1.18
*p*	0.8526	0.1447	> 0.999	0.3861	0.4645	0.6829	> 0.999	0.8009	0.5886	0.4642	> 0.999	0.0854

*Note:* The *p* value was obtained by comparing each group after 6 months of treatment with the first treatment.

### 3.7 Patient Satisfaction

Following the comprehensive facial anti‐scarring treatment, patients were asked to complete a satisfaction questionnaire. The results indicated high satisfaction: 15 cases (68.18%) reported being very satisfied, 3 cases (13.64%) were satisfied, and another 3 cases (13.64%) reported being more satisfied. One case (4.54%) indicated general satisfaction (Table 7).

**TABLE 7:** Patient satisfaction with the transparent mask.

Patient satisfaction	Number	Percentage
Very satisfied	15	68.18
Satisfied	3	13.64
More satisfied	3	13.64
Generally satisfied	1	4.54
Not very satisfied	0	0
Dissatisfied	0	0
Very dissatisfied	0	0

### 3.8 Adverse Effects

During the period of wearing the transparent mask, 2 cases (9.09%) of redness, 1 case (4.55%) of oozing, 2 cases (9.09%) of blistering, and 1 case (4.55%) of rash were observed. Among them, there were 2 cases (9.09%) with two or more adverse effects, and 14 cases (63.64%) without any adverse effects (Table 8).

**TABLE 8:** Adverse effects of wearing transparent masks.

Adverse effects	Number	Percentage
Redness	2	9.09
Oozing	1	4.55
Bleeding	0	0
Blistering	2	9.09
Swelling	0	0
Rash	1	4.55
Two and more	2	9.09
Without adverse effects	14	63.64

## 4 Discussion

Deep facial burns with second degree or third degree usually leave conspicuous scars which not only affect the normal facial appearance, but also cause facial deformation and development due to scar contracture, both of which bring physical and psychological trauma to the patient. Currently, pressure therapy is one of the mainstay treatments for hypertrophic scars. The rigid transparent mask made from thermoplastic materials overcomes the shortcomings of traditional masks by exerting effective pressure on concave surfaces, offering long‐lasting preventive and therapeutic effects. Benefits include reducing hypertrophic scar formation, maintaining facial contours, promoting smoother scar surfaces, facilitating facial fixation, and allowing easy observation due to transparency.

Although the application of pressure therapy using transparent masks has been practiced for almost 40 years, evidence supporting its clinical effectiveness remains limited. One study found that scar thickness decreased at 1 and 3 months after facemask application, but it was not significant [4]. A survey in North America found that 87% of burn patients were routinely treated with a transparent mask for facial scarring [5]. And Koudougou reported that the treatment was well‐tolerated in patients, particularly in the degree of scar color and thickness improvement [6]. A follow‐up evaluation of the transparent masks revealed a decrease in average scar thickness and a significant improvement in facial appearance [7]. The scar thickness after 1‐month mask treatment, especially on the forehead, eyes, nose, and mouth site, showed a positive linear relationship between changes for scar thickness and the pressure applied locally [8]. In terms of its treatment mechanism, studies have demonstrated that the effectiveness of transparent masks in the treatment of hypertrophic scar is achieved through two avenues: (1) the transparent mask conforms to the majority of facial curves and contours, and (2) the transparent mask applies appropriate pressure on the facial scar [9]. However, there is no consensus on the optimal pressure needed to achieve satisfactory results. Candy reported that pressure therapy should be at least 24–25 mmHg to overcome capillary pressures within the scar tissue [10]. Elsewhere, low doses of pressure seemed to provide acceptable and effective healing [11, 12]. In addition, the recommended period of wearing the facemask varies across studies [10, 13]. The 2016 ISBI guidelines recommend that transparent masks should be subjected to a pressure of 20–32 mmHg for 20–23 h per day for at least 2 months and up to 2 years [14].

**FIGURE 1:**
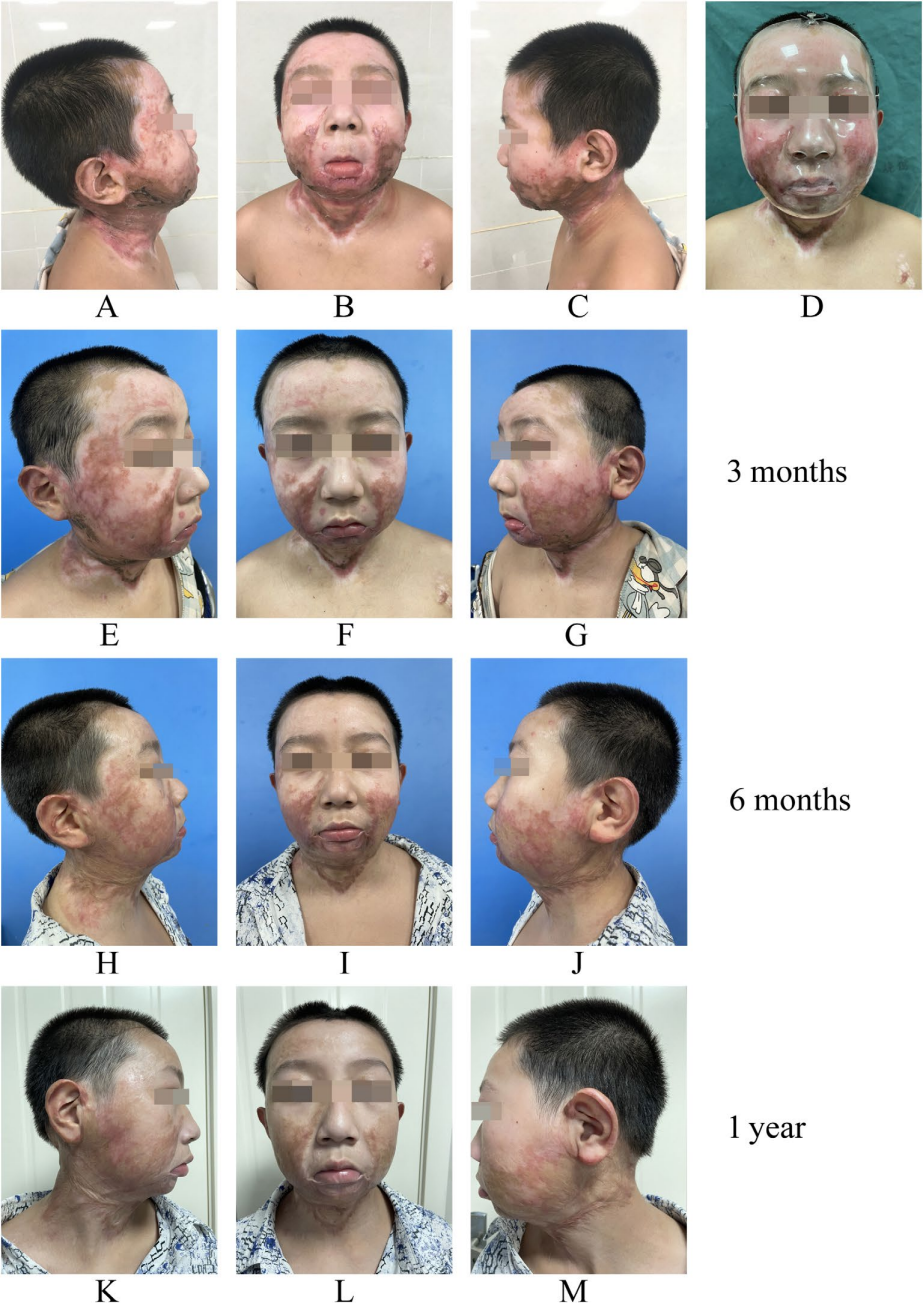
Efficacy of the transparent mask on facial hypertrophic scar after burns. A 10‐year‐old male patient accidentally suffered flame burns to his face and neck with a total burn area of 10% TBSA and a third‐degree facial burn. He received topical medication and autologous skin grafting. And the patient wore a transparent pressure mask after 2 months post‐burn. (A) Patient's first assessment (right side face). (B) Patient's first assessment (frontal face). (C) Patient's first assessment (left side face). (D) Patient wearing transparent masks. (E) After 3 months of wearing a transparent mask (right side face). (F) After 3 months of wearing a transparent mask (frontal face). (G) After 3 months of wearing a transparent mask (left side face). (H) After 6 months of wearing a transparent mask (right side face). (I) After 6 months of wearing a transparent mask (frontal face). (J) After 6 months of wearing a transparent mask (left side face). (K) After 1 year of wearing a transparent mask (right side face). (L) After 1 year of wearing a transparent mask (frontal face). (M) After 1 year of wearing a transparent mask (left side face).

Although the rigid transparent mask can provide pressure on the concave surface, there was no evidence to compare the treatment outcome of facial hypertrophic scar on convex and concave surface. In our study we for the first time found the VSS scores of the middle of the forehead and bilateral zygomatic arches decreased more than that of bilateral cheeks and chin after six mask treatments, which indicated that the therapeutic effect of the transparent mask on convex scar was better than that on concave scar. Notably, mask wearing for 6 months yielded significant improvements in scarring; the facial scars exhibited a gradual maturation process, appearing generally well‐controlled with minimal distortion to facial appearance (Figure 1). However, the VAS pruritus and pain scores in the middle of forehead, bilateral cheeks, and chin regions showed a decreasing trend, whereas the VAS pain scores in the bilateral zygomatic arches showed an increasing trend after 6 months with no significant results. This phenomenon may be ascribed to the pressure‐induced pain on the scar generated by the transparent mask and prolonged wearing of a single transparent mask. Furthermore, in our study we also analyzed the adverse effects of wearing masks and found 8 out of the 22 patients developed adverse effects during the entire treatment period, accounting for 36.36% of the total number of patients. The efficacy of the transparent mask is partly dependent on the application of sufficient pressure. Some adverse effects such as redness, oozing, blistering, and rash may occur during pressurization, which may worsen during the extended mask wearing period. Therefore, considering the remodeling of the scar and the occurrence of adverse effects, routine mask modifications are necessary to ensure proper wearing and maintain sufficient pressure.

In addition to the subjective assessment, we also added objective devices including ultrasound, durometer, and spectrocolorimeter to analyze the thickness, hardness, and color of scar in this study. After six mask treatments, we found that the thickness of the six sites exhibited a decreasing trend after 6 months, but only the mid‐forehead and chin had a significant difference, which indicated that the transparent mask, when applied to the protruding area of the middle of the forehead, exerts greater pressure and has a more significant effect. Interestingly, only the hardness of the chin scar had a significant decrease, although the other five sites showed a decreasing trend in the scores. Spectrocolorimeter is employed as a gold standard to measure the scar pigmentation and vascularity in terms of lightness, redness, and yellowness [15]. A significant difference was observed in the *L* value of scars on the mid‐forehead, left zygomatic arch, right cheek, and chin after mask use, indicating a statistically significant improvement in scar coloration. These objective results were consistent with the subjective results from VSS scores, which indicated that the transparent mask had a better effect on facial protrusion scar.

## 5 Limitations of the Study and Future Perspectives

The follow‐up duration for the patients was relatively short. It is better to extend the observation period until the hypertrophic scar mature. Because of the high cost of mask production, the patients usage rate is low, resulting in a smaller number of patients in our study. Although the available evidence supporting the clinical effects of transparent masks is limited, it is the only modality that uses mechanical compression to treat severe burn‐related facial hypertrophic scar. Therefore, it remains the mainstay method for treating severe facial scar.

## 6 Conclusion

In this study, the transparent mask prepared through the traditional plaster molding exerted sufficient compression pressure within 1 month to yield favorable clinical effects. Specifically, it improved facial hypertrophic scar especially in the middle of forehead and bilateral zygomatic arches sites, and reduced the symptoms of scar such as pruritus and pain, while also reducing facial distortion and accelerating the scar maturation. Future studies with larger sample sizes are advocated to verify these conclusions and promote the widespread application.

## Funding

This work was supported by the National Natural Science Foundation of China (82072188) and the Independent Research Project of State Key Laboratory of Trauma and Chemical Poisoning (SKLZZ201802).

## Ethics Statement

This retrospective analysis was approved by the Ethics Committee of the First Affiliated Hospital of Army Medical University.

## Consent

The authors declare they agree to submit this manuscript.

## Conflicts of Interest

The authors declare no conflicts of interest.

## Data Availability

The data that support the findings of this study are available on request from the corresponding author. The data are not publicly available due to privacy or ethical restrictions.
